# Effects of an immersive virtual reality reminiscence intervention on engagement, behavioral and psychological symptoms, and well-being of people with dementia: A randomized crossover trial

**DOI:** 10.1177/13872877251371236

**Published:** 2025-08-29

**Authors:** Miguel Pereira, Cláudia Leite, Carlos Campos, Tiago Coelho

**Affiliations:** 1 203255 E2S, Polytechnic of Porto, Porto, Portugal; 2 386292 Lusófona University, HEI-Lab: Digital Human-Environment Interaction Labs and LabRP/CIR, E2S, Polytechnic of Porto, Porto, Portugal; 3 203255 LabRP/CIR, E2S, Polytechnic of Porto, Porto, Portugal

**Keywords:** Alzheimer's disease, behavioral and psychological symptoms, dementia, engagement, heart rate variability, reminiscence therapy, virtual reality, well-being

## Abstract

**Background:**

Virtual reality (VR) is a novel technology that can facilitate reminiscence in people with dementia. However, few studies have explored the role of VR's immersiveness in enhancing therapeutic outcomes.

**Objective:**

This study aimed to analyze the effects of an immersive VR reminiscence session compared to a non-immersive session, focusing on engagement, behavioral and psychological symptoms (BPSD), and well-being, using behavioral observation and physiological metrics.

**Methods:**

A randomized crossover trial with a seven-day washout period was conducted. Engagement, BPSD and well-being were assessed before, during and after each intervention using both observational scales and heart rate variability analysis.

**Results:**

20 participants (average age 80.55 years, 90% women) were recruited. Significant pre-post differences were found in behavioral engagement (z = −2.67, p = 0.008) and facial expression of apathy (z = −2.12, p = 0.034) during the immersive intervention. Additionally, lower apathy in terms of purposeful activity was observed during the immersive intervention (z = −2.24, p = 0.025). These results are particularly noteworthy given the sample size, which, although small, highlights a clear trend of behavioral and apathy change.

**Conclusions:**

The results suggest that VR's immersiveness can enhance engagement in dementia intervention programs. As VR technology becomes more accessible and safer, continued research is needed to explore its therapeutic potential.

## Introduction

Dementia is a progressive neurodegenerative syndrome marked by a decline in cognitive skills and daily functioning, often accompanied by behavioral and psychological symptoms in dementia (BPSD),^1,2^ such as aggression, agitation, apathy, and depression.^3,4^ These symptoms negatively impact the quality of life (QoL) and well-being of people with dementia and their families.^5,6^ In 2023, it was estimated that 55 million people worldwide had dementia, with over 60% of them residing in low- or middle-income countries, and around 10 million new cases annually.^7^ Dementia is the seventh leading cause of mortality worldwide and significantly contributes to dependency and disability among older adults.^7^

Non-pharmacological therapies are recommended as first-line treatments for BPSD,^8,9^ as traditional medications can lead to adverse effects, including increased fall risk and mortality.^10,11^ According to recent systematic reviews, various non-pharmacological interventions, such as sensory stimulation, cognitive therapies, and behavioral management techniques, have been shown to reduce BPSD and improve cognitive function, daily living activities, and social interaction,^12,13^ ultimately enhancing independence and increase QoL.^6,14^

Non-pharmacological interventions have also been studied as beneficial to the well-being of people with dementia,^6,15^ primarily assessed through self-report scales and observation, with some studies measuring physiological metrics that translates into improvements in well-being by analyzing heart rate variability (HRV) and the autonomic nervous system (ANS).^16,17^ These metrics, though less commonly used, offer deeper insight into the effects of such interventions on brain activity and emotional states.^18^

Reminiscence therapy (RT) was introduced to dementia care in the late 1970s^19,20^ and utilizes materials such as photographs, familiar items from the past and music to evoke memories and encourage people to share and cherish their past experiences, enhancing well-being.^12,21^ RT has been demonstrated to be beneficial for managing BPSD,^22,23^ such as depression, anxiety and mood.^24,25^ It leverages individuals’ stronger recall of early life events, promoting engagement in therapy and self-identity.^21,26^

Digital technologies have introduced innovative reminiscence tools, notably virtual reality (VR), which can enhance memory recall and encourage reminiscence through immersive experiences.^27,28^ VR is a term that refers to the set of hardware and software components that replicate a simulated environment, enabling users to interact with three-dimensional computer-generated environments or 360° video footage.^29^ VR comes in different forms, ranging from fully immersive systems to non-immersive. Non-immersive systems frequently use a computer monitor to interact with the virtual environment.^30^ Systems that offer completely immersive experiences include the user feeling physically present within a virtual environment using a head-mounted display (HMD) or a Cave, by reducing real-life stimuli.^30^

According to recent studies, immersive VR is safe, well-tolerated, and able to encourage engagement and give dementia patients enjoyable experiences.^28,31^ Also, Rose et al.'s study revealed that dementia participants frequently reminisced and spontaneously reenacted stories from their past when they watched 360° videos of places that appeared familiar and important to each person's life experience.^29^

With inconsistent findings, numerous studies have also investigated the efficacy of VR for decreasing BPSD and enhancing the QoL for dementia patients.^32,33^ In particular, some VR reminiscence studies observed that this intervention can be feasible,^34^ safe,^35^ well-accepted^36^ and promotes positive levels of comfort, pleasure, satisfaction with the experience,^37^ and engagement to social interactions.^38^

Despite these promising benefits, in the context of dementia care, VR reminiscence interventions are novel and under researched.^36^ Moreover, most studies that evaluate the effect of RT or RT using VR on people with dementia evaluate using questionnaires and behavioral measures, with few studies evaluating people's physiological metrics during the intervention.^36,39^ With all this in mind, the aim of this study was to analyze the effect of an immersive VR reminiscence session compared to a non-immersive reminiscence session, in terms of engagement, BPSD and well-being in people with dementia, using behavioral observation and measurement of physiological metrics during interventions.

Considering the objective of the present study, the following hypothesis was determined for this study:

H1: The application of a reminiscence intervention using immersive VR (HMD) contributes to a greater engagement, less BSPD and to a greater well-being in session compared to a non-immersive reminiscence intervention (monitor), using 360° videos from locations relevant to the participants.

## Methods

### Recruitment

This study was a randomized crossover trial, with each participant undergoing two interventions: an immersive VR reminiscence session and a non-immersive reminiscence session, separated by a seven-day washout period.^40^ Which intervention each participant performed first was randomized.

Ethics approval was obtained from the E2S – Polytechnic of Porto (CE0031E). Institutions were contacted via email to recruit participants, and consent was secured from the participant or the participants’ families, adhering to the Declaration of Helsinki.^41^ The consent form outlined the study's objectives, procedures, confidentiality measures, and participants’ rights to withdraw at any time without penalty.

**Figure 1.**
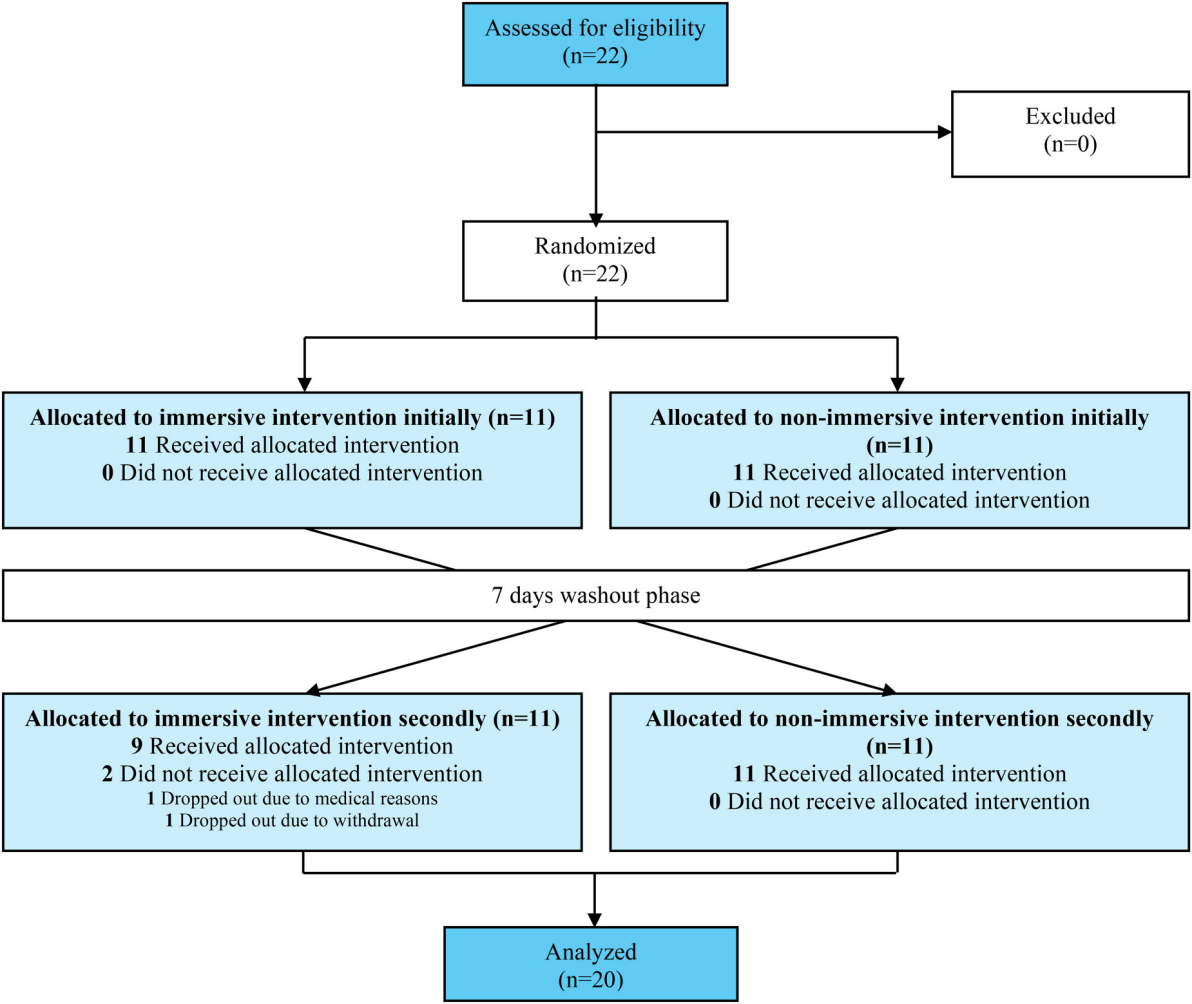
CONSORT flow diagram.

Participants were recruited through non-probabilistic convenience sampling from dementia care institutions accessible to researchers. A sample of 22 participants was recruited considering as inclusion criteria having diagnosis of dementia. Considered exclusion criteria were: severe visual deficits that prevented them from experiencing the videos, severe communication deficits that do not allowed them to express their life stories, as well as describe their sensations during the intervention, score of 7 on the Global Deterioration Scale (GDS)^42^ which represents an advanced stage of the dementia process, having a diagnosis of Lewy body type dementia due to the likelihood of visual hallucinations, and having severe motor limitations that prevented the active exploration of the videos. Within this sample of participants, 11 began the intervention with an immersive approach and 11 began with a non-immersive approach. However, 2 participants were excluded from the study after it was started, as shown in Figure 1.

### Measurements

The level of dementia progression was measured with the Global Deterioration Scale (GDS).^42,43^ The Engagement of a Person with Dementia Scale (EPWDS)^44^ was used to assess engagement during the session, the Person-Environment Apathy Rating (PEAR)^45^ was used to assess apathy, a commonly BPSD, before, during and after the session, the Observed Emotion Rating Scale (OERS)^46^ was used to assess BPSD during the session, such as pleasure, anger, anxiety/fear, sadness and general alertness, the Observable Well-Being in Living With Dementia Scale (OWLS)^47^ was used to assess well-being during the session. Furthermore, a questionnaire was developed to characterize the participants regarding sex, birth date, marital status and education years. After each session, to evaluate the participant's self-perception of the experience, a Likert scale from 1 to 5 was also created with the following questions: “Did you like the experience?”, “How was the experience?”, “How motivated are you to do this activity again?”, “Interested in seeing other places?”, “How well did you manage to watch the video?” and “How comfortable was the activity?”. To analyze the psychophysiological metrics, electrocardiography (ECG)^48^ was used to assess engagement and well-being during the session, through the HRV and ANS activity.

The GDS^42^ is a scale that identifies the stage of degenerative dementia and encompasses functional and cognitive deficits. It is a seven-level rating scale in which level 1 reflects no cognitive decline, level 2 reflects very mild cognitive decline, and levels 3 through 7 are defined, respectively, as mild, moderate, moderately severe, severe, and very severe cognitive impairment. Each level is associated with clinical stages ranging from normal (level 1) to late-onset dementia (level 7). This scale is completed with the help of professionals from the institutions.

The EPWDS^44^ is a scale that assesses and measures the degree of engagement of participants during interventions. It consists of five categories of engagement: affective, visual, verbal, behavioral and social engagement. Each category is divided into two items, one of which is perceived as positive involvement and the other as negative involvement. The total score varies from ten to fifty points, and the higher the total score, the greater the positive engagement demonstrated by the person. Each item is scored according to a five-point Likert scale (from 1 to 5). In terms of internal consistency, the Cronbach's alpha is 0.94.^44^

The PEAR^45^ is a scale used to assess participants’ apathy before, during and after sessions. Apathy is measured by looking at six indicators: facial expressions, eye contact, physical engagement, purposeful activity, verbal tone and verbal expression, on a scale of 1 to 4, with higher scores indicating a greater level of apathy. This scale can also be an aid to assess engagement based on apathy levels. In terms of internal consistency, the Cronbach's alpha is 0.85 for the PEAR-Apathy subscale version.^45^

The OERS^46^ is an observational scale to assess two positive emotions (pleasure and general alertness) and three negative emotions (anger, anxiety or fear, and sadness). During a 10-min period, the researcher chooses one of six possible defined time intervals (e.g., 1 = never; 2 = <16 s; 3 = 16–59 s; 4 = 1–5 min; 5 = >5 min; and 7 = not in sight) that a target subject demonstrates each of the five emotions. Higher scores indicate longer duration of expression of that emotion.

The OWLS^47^ is an instrument used to code observed expressions of well-being in people with dementia when participating in activities. The person is observed for 30 s. All items are scored dichotomously as “1” if present and “0” if not present. It consists of eight categories of well-being: attention, initiative/responsiveness, relaxation, happiness, pleasure, expressions of identity, mastery and relationships. For each category, a scale of 1–10 is used to evaluate the relative frequency during the 30-s intervals. The higher the score, the higher the levels of well-being. In terms of internal consistency, the inter-rater reliability's Cohen's Kappa is 0.82 and the intra-rater reliability's Cohen's Kappa is 0.98.^47^

Heart rate variability (HRV) data were recorded using electrocardiography (ECG),^48^ assessing emotional activation through time-domain parameters like: Mean HR, since its variability is closely related to emotional activation^48,49^; SDNN, which is the standard deviation of all R-R intervals, to analyze the cyclic components responsible for HRV; RMSSD, described as the root mean square of successive differences, to analyze parasympathetic nervous system (PNS); pNN50, which is the percentage of successive normal sinus RR intervals more than 50 ms, to also analyze PNS.^49-52^ In terms of Frequency Domain (FFT Welch Method) on Low frequencies (LF) (0.04 Hz, 0.15 Hz), to analyze the mix of sympathetic and parasympathetic activity and the baroreflex activity, and High frequencies (HF) (0.15 Hz, 0.4 Hz), to analyze the PNS, were used: Peak Frequencies and Absolute Powers.^49-52^ Moreover, the Total Power, which is the sum of the energy in the LF and HF bands, and LF/HF Ratio, which is the ratio between sympathetic nervous system (SNS) and PNS, were also analyzed.^53^ On Non-linear indices were used: SD1, which is the standard deviation – Poincaré plot Crosswise, to analyze quick and high frequent changes in HR variability; SD2, which is the standard deviation – Poincaré plot Lengthwise, to analyze long-term changes in HRV.^49-52^

### Intervention

Prior to data collection, the evaluation protocol and methods were established by the research team. Authorization for the assessment scales was obtained from the respective authors, and translated versions were utilized for outcome evaluation. Researchers received training on the protocol, followed by a pilot evaluation phase involving relatives and older adults to test its effectiveness. Adjustments were made based on pilot feedback before initiating sessions with participants.

In the pre-intervention session, participants and their caregivers identified a significant location from a pre-filmed list, and the characterization questionnaire was completed. Researchers confirmed this information with caregivers to ensure accuracy. To maintain confidentiality, each participant was assigned an alphanumeric code. During this period, institutional professionals completed the GDS for each participant.

Regarding the intervention, each participant was exposed to two sessions, separated by a seven-day washout period. One session involved viewing a significant location video using the *Oculus Quest 2* HMD, after a demonstration of the device. Participants first viewed a neutral video (library) for 2–3 min to familiarize themselves with the HMD, followed by the significant location video for 5–6 min. In the other session, the same intervention structure was used, using a computer monitor and a mouse to explore it. There was randomization of participants regarding which session they would perform first, carried out by a researcher not involved in data collection and intervention. Sessions lasted 20–30 min and took place in the participant's preferred setting between May and July 2024.

During exposure to the two experimental conditions, the participant's peripheral physiological activity was recorded using the BITalino Core BT/BLE amplifier (acquisition frequency 1000 Hz). More specifically, the heart rate (HR) and HRV were collected through ECG with 3 electrodes placed on the trunk (positive electrode on the right clavicle; negative electrode on the left iliac crest; ground electrode on the clavicle left). The procedure consists of a non-invasive, painless collection with no known side effects. To apply the electrodes, it was necessary to clean the area of the skin where the electrodes were applied with alcohol and abrasive gel, to remove any residue that could interfere with collection. The electrodes were subsequently fixed using tape (adhesive tape suitable for application to the skin). Precautions were only necessary when cleaning and preparing the skin of participants with skin problems and/or open wounds in the positions where the electrodes were to be placed. After placing the electrodes, the participant was asked to remain relaxed and silent for 5 min so that their physiological signals could return to a normal state. After 5 min, physiological signals were recorded at rest for 2 min. Physiological signals were also recorded throughout the period of habituation and exposure to the significant location video.

Various assessment instruments were applied before, during, and after the sessions to evaluate engagement, BPSD, and well-being. During the sessions, in both interventions, the researchers asked several questions, following a previously defined guide, to allow greater discussion and communication with the participant. With the aim of discussing the observed behaviors with the entire research team and the researchers present could focus on the parameters of the protocol and the personal stories that the participants were describing, the session was filmed, in accordance with the consent of the participant and caregivers. The camera was placed in front of the participant to capture their entire body and all their movements and reactions. Filming captured participants from entry to exit. After each session, the participant's self-perception of the experience scale was applied. Data collection was carried out by two properly trained researchers who followed the same data collection protocol.

### Statistical analysis

Statistical analysis was performed using the IBM Statistical Package for the Social Science (SPSS) version 29 software. First, descriptive statistics were used to present the characteristics of the participants and the instruments results. According to the nature of the variables, categorical variables were presented as number (n) and percentages (%) and continuous variables were presented with the mean (M), standard deviation (SD), minimum and maximum values.

Subsequently, to be able to analyze the differences between the two interventions, immersive and non-immersive, through the results of the different scales, the paired samples t-test was applied, whenever the normality of the variables was assumed. When this assumption was not assumed, the respective non-parametric test, the Wilcoxon signed-rank test, was applied.

The statistical assumptions for carrying out the tests were validated through the normality analysis of the Shapiro-Wilk test variables, with the normal distribution of variables being assumed whenever the p-value was greater than the significance level. A significant level of 0.05 was considered when carrying out all statistical tests.

Regarding ECG data, data preprocessing and measure extraction was conducted using the pyHRV Python Toolbox pipeline. Processing of ECG included signal filtering (3 Hz–45 Hz), R-peak detection (Hamilton-Tompkins algorithm) and lastly feature extraction.^54,55^ Outliers were removed using the 3 interquartile range criteria^56^ and the statistical significance threshold was set at 0.05. Descriptive and inferential statistics were computed using SPSS. For inferential statistics, parametric tests were used whenever data distribution was deemed approximately normal using threshold criteria for skewness and kurtosis – less than |2.0| and |9.0|, respectively.^56^ Additionally, two-way repeated-measures ANOVAs were implemented for comparing the three conditions in the two types of interventions. For these models, sphericity was tested using Mauchly's test. The Huynh-Feldt correction was employed whenever this assumption was not met and the epsilon was higher than 0.57; otherwise, the Greenhouse-Geisser correction was used.^56^

## Results

### Sample characteristics

According to Table 1, the study included 20 participants, aged between 68 and 93 (M = 80.55 ± 6.69), the majority were female (n = 18, 90%) and 11 participants were widows (55%). Regarding education, the average number of years spent studying by participants was 4.75 years (SD ± 2.07). The most common level of deterioration, classified by the GDS, was moderate cognitive decline (n = 10, 50%), with a mean scale score of 4.15 (SD ± 0.81). For more detailed information, consult Table 1.

**Table 1.** Sample characteristics, through sociodemographic variables and dementia stage, through the GDS scores.

	**Sample**
	**n = 20**
	**n (%)**
**Age (years)**	
** Mean ± Standard deviation**	80.55 ± 6.69
** Min-Max**	68–93
**Sex**	
** Feminine**	18 (90)
** Masculine**	2 (10)
**Education (years)**	
** Mean ± Standard deviation**	4.75 ± 2.07
** Min-Max**	0–9
**Marital status**	
** Married**	6 (30)
** Widow/er**	11 (55)
** Single**	2 (10)
** Divorced**	1 (5)
**GDS**	
** Mild cognitive decline**	4 (20)
** Moderate cognitive decline**	10 (50)
** Moderately severe cognitive decline**	5 (25)
** Severe cognitive decline**	1 (5)
**GDS score (1–7)**	
** Mean ± Standard deviation**	4.15 ± 0.81

### Comparison of engagement in the different interventions

In terms of engagement in the session, the results indicated that there was no significant difference between the immersive intervention and non-immersive intervention, t (19) = 1.058, p = 0.303. Analyzing the mean EPWDS scores, although there were no significant differences, considering the maximum EPWDS score, the mean obtained in the two interventions indicates that the engagement in both sessions was positive (see Supplemental Table 1). According to Table 2, the results indicated that the behavioral engagement in session was significantly higher on immersive intervention (M = 4.33, SD = 0.52) compared to non-immersive intervention (M = 4.05, SD = 0.58), z = −2.67, p = 0.008. Regarding all the remaining engagement indicators on the EPWDS scale, it was possible to conclude that there was no significant difference between the immersive and non-immersive intervention.

**Table 2.** Comparison of the immersive and non-immersive intervention according to the EPWDS categories scores.

Mean ± Standard deviation			
	Immersive intervention	Non-immersive intervention	p
Affective engagement	4.20 ± 0.71	4.23 ± 0.67	0.816^a^
Visual engagement	4.70 ± 0.38	4.63 ± 0.39	0.499^a^
Verbal engagement	4.55 ± 0.39	4.58 ± 0.44	0.813^a^
Behavioral engagement	4.33 ± 0.52	4.05 ± 0.58	0.008^a^
Social engagement	4.23 ± 0.47	4.13 ± 0.53	0.356^a^

^a^ value obtained using the Wilcoxon signed-rank test.

### Comparison of behavioral and psychological symptoms in the different interventions

According to Table 3, the results indicated that apathy on facial expressions during the session was significantly higher on immersive intervention (M = 2.30, SD = 0.80) compared to non-immersive intervention (M = 2.00, SD = 0.65), z = −2.12, p = 0.034. It also indicated that apathy on purposeful activity during the session was significantly higher on non-immersive intervention (M = 1.75, SD = 0.72) compared to immersive intervention (M = 1.50, SD = 0.51), z = −2.24, p = 0.025. Regarding all the remaining apathy indicators on the PEAR scale, it was possible to conclude that there was no significant difference between the immersive and non-immersive intervention. In terms of pleasure (z = −0.25, p = 0.803), anger (z = 0.00, p = 1.000), anxiety or fear (z = −1.00, p = 0.317), sadness (z = −0.38, p = 0.705) and general alertness (z = 0.00, p = 1.000) in the session, the results indicated that there was no significant difference between the immersive and non-immersive intervention (see Supplemental Table 2).

**Table 3.** Comparison of the immersive and non-immersive intervention according to the PEAR scores.

		Mean ± Standard deviation		p
		Immersive intervention	Non-immersive intervention	
Before	Facial expressions	2.85 ± 0.88	2.85 ± 0.88	1.000^a^
	Eye contact	1.45 ± 0.69	1.40 ± 0.69	0.763^a^
	Physical engagement	2.80 ± 0.95	2.85 ± 0.88	0.796^a^
	Purposeful activity	2.45 ± 1.15	2.40 ± 1.14	0.785^a^
	Verbal tone	2.85 ± 0.75	2.90 ± 0.85	0.763^a^
	Verbal expression	2.40 ± 0.75	2.15 ± 0.88	0.059^a^
During	Facial expressions	2.30 ± 0.80	2.00 ± 0.65	0.034^a^
	Eye contact	1.35 ± 0.49	1.20 ± 0.41	0.180^a^
	Physical engagement	1.80 ± 0.83	2.00 ± 0.86	0.248^a^
	Purposeful activity	1.50 ± 0.51	1.75 ± 0.72	0.025^a^
	Verbal tone	2.25 ± 0.55	2.20 ± 0.52	0.655^a^
	Verbal expression	1.65 ± 0.59	1.50 ± 0.51	0.257^a^
After	Facial expressions	2.40 ± 0.75	2.55 ± 0.61	0.405^a^
	Eye contact	1.25 ± 0.44	1.20 ± 0.41	0.655^a^
	Physical engagement	2.35 ± 0.88	2.35 ± 0.88	1.000^a^
	Purposeful activity	1.95 ± 0.63	1.85 ± 0.67	0.595^a^
	Verbal tone	2.30 ± 0.66	2.40 ± 0.50	0.516^a^
	Verbal expression	1.80 ± 0.70	1.90 ± 0.64	0.480^a^

^a^ value obtained using the Wilcoxon signed-rank test.

### Comparison of well-being in the different interventions

The results indicated that there was no significant difference between the immersive and non-immersive intervention in terms of well-being in the session. Analyzing the mean OWLS scores, although there was no significant difference, a tendency towards a higher level of happiness and pleasure in immersive intervention compared to non-immersive intervention may emerge. It can also be observed that in both interventions all participants presented themselves attentive, responsive and relaxed during the intervention (see Supplemental Table 3).

### Comparison of physiological metrics in the different interventions

Relatively to the time domain results (see Supplemental Material), for the repeated-measures ANOVA for mean HR, the sphericity assumption was not met (p < 0.001) and the epsilon was 0.618 for the condition effect and 0.589 for the condition * intervention interaction. Thus, the Huynh-Feldt correction was employed for this model. The results indicated a significant main effect for condition, F (1.235,19.765) = 4.333, p = 0.043, partial η2 = 0.213. Post-hoc pairwise comparisons with a Bonferroni correction indicated that mean HR in the baseline rest condition (M = 67.55, SD = 2.58) was significantly lower (p = 0.020) than in the exposure condition (M = 69.79, SD = 2.31). However, there was no significant main effect for intervention, F (1,16) = 0.026, p = 0.875, partial η2 = 0.002, and non-significant intervention*condition interaction, F (1.178,18.852) = 0.279, p = 0.641, partial η2 = 0.017.

The repeated-measures ANOVA results for SDNN indicated no significant main effect for intervention, F (1,16) = 0.069, p = 0.796, partial η2 = 0.004, condition, F (2,32) = 2.501, p = 0.098, partial η2 = 0.135 and intervention*condition interaction, F (2,32) = 0.256, p = 0.776, partial η2 = 0.016.

For the repeated-measures ANOVA for RMSSD, the threshold criteria for skewness and kurtosis assumption was not met (skewness > 2), despite being very close values. The results indicated no significant main effect for intervention, F (1,16) = 0.007, p = 0.935, partial η2 = 0.000, condition, F (2,32) = 1.729, p = 0.194, partial η2 = 0.098 and intervention*condition interaction, F (2,32) = 0.458, p = 0.637, partial η2 = 0.028.

The repeated-measures ANOVA results for pNN50 indicated no significant main effect for intervention, F (1,19) = 1.466, p = 0.241, partial η2 = 0.072, condition, F (2,38) = 0.006, p = 0.994, partial η2 = 0.000 and intervention*condition interaction, F (2,38) = 0.611, p = 0.548, partial η2 = 0.031.

Considering the frequency domain results (see Supplemental Material), the repeated-measures ANOVA results for Peak Frequencies on LF band indicated no significant main effect for intervention, F (1,19) = 0.112, p = 0.741, partial η2 = 0.006, condition, F (2,38) = 0.857, p = 0.433, partial η2 = 0.043 and intervention*condition interaction, F (2,38) = 0.076, p = 0.927, partial η2 = 0.004. In terms of Peak Frequencies on HF band, the repeated-measures ANOVA results indicated no significant main effect for intervention, F (1,19) = 0.297, p = 0.592, partial η2 = 0.015, condition, F (2,38) = 0.480, p = 0.623, partial η2 = 0.025 and intervention*condition interaction, F (2,38) = 0.182, p = 0.834, partial η2 = 0.010.

For the repeated-measures ANOVA for Absolute Powers on LF band, the threshold criteria for skewness and kurtosis assumption was not met (skewness > 2 and kurtosis > 9). The sphericity assumption was not met for the condition * intervention interaction (p < 0.001, epsilon = 0.576). Thus, the Huynh-Feldt correction was employed for this model. The results indicated no significant main effect for intervention, F (1,14) = 0.029, p = 0.866, partial η2 = 0.002, condition, F (2,28) = 0.465, p = 0.633, partial η2 = 0.032 and intervention*condition interaction, F (1.153,16.140) = 0.231, p = 0.672, partial η2 = 0.016. For the repeated-measures ANOVA for Absolute Powers on HF band, the threshold criteria for skewness and kurtosis assumption was not met (skewness > 2 and kurtosis > 9). The sphericity assumption was not met for the condition effect (p < 0.001, epsilon = 0.594) and for the condition * intervention interaction (p < 0.001, epsilon = 0.547). Thus, the Huynh-Feldt correction was employed for the condition effect and the Greenhouse-Geisser correction was employed for the condition * intervention interaction. The results indicated no significant main effect for intervention, F (1,14) = 1.386, p = 0.259, partial η2 = 0.090, condition, F (1.187,16.619) = 2.493, p = 0.130, partial η2 = 0.151 and intervention*condition interaction, F (1.094,15.321) = 0.449, p = 0.530, partial η2 = 0.031.

For the repeated-measures ANOVA for Total Power, the threshold criteria for skewness and kurtosis assumption was not met (skewness > 2 and kurtosis > 9). The sphericity assumption was not met for the condition effect (p = 0.014, epsilon = 0.732). Thus, the Huynh-Feldt correction was employed for the condition effect. The results indicated no significant main effect for intervention, F (1,15) = 0.460, p = 0.508, partial η2 = 0.030, condition, F (1.463,21.948) = 0.295, p = 0.679, partial η2 = 0.019 and intervention*condition interaction, F (2,30) = 0.471, p = 0.629, partial η2 = 0.030.

For the repeated-measures ANOVA for LF/HF Ratio, the threshold criteria for skewness and kurtosis assumption was not met (skewness > 2). The results indicated no significant main effect for intervention, F (1,13) = 0.272, p = 0.611, partial η2 = 0.020, condition, F (2,26) = 0.015, p = 0.985, partial η2 = 0.001 and intervention*condition interaction, F (2,26) = 0.739, p = 0.488, partial η2 = 0.054.

In terms of the non-linear indices results (see Supplemental Material), for the repeated-measures ANOVA for SD1, the threshold criteria for skewness and kurtosis assumption was not met (skewness > 2). The results indicated no significant main effect for intervention, F (1,16) = 0.007, p = 0.935, partial η2 = 0.000, condition, F (2,32) = 1.729, p = 0.194, partial η2 = 0.098 and intervention*condition interaction, F (2,32) = 0.458, p = 0.637, partial η2 = 0.028. The repeated-measures ANOVA results for SD2 indicated no significant main effect for intervention, F (1,16) = 0.124, p = 0.729, partial η2 = 0.008, condition, F (2,32) = 2.858, p = 0.072, partial η2 = 0.152 and intervention*condition interaction, F (2,32) = 0.171, p = 0.843, partial η2 = 0.011. In terms of SD2/SD1 Ratio, the repeated-measures ANOVA results indicated no significant main effect for intervention, F (1,18) = 0.177, p = 0.679, partial η2 = 0.010, condition, F (2,36) = 0.970, p = 0.389, partial η2 = 0.051 and intervention*condition interaction, F (2,36) = 0.086, p = 0.918, partial η2 = 0.005.

### Comparison of the participant's self-perception of the experience in the different interventions

The participant's self-perception of the experience questionnaire results (see Supplemental Table 9) demonstrated that generically the participants enjoyed both interventions (immersive and non-immersive), with a tendency to evaluate more positively to the immersive intervention than non-immersive intervention, despite reporting slightly greater difficulties to visualize the videos in the immersive intervention.

The results indicated that there was no significant difference between the immersive and non-immersive intervention in terms of the participant's self-perception of the experience in the session. Analyzing the questionnaire mean scores, although there were no significant differences, a tendency to enjoy more immersive intervention compared to non-immersive may emerge. Also, a tendency to watch the video better on non-immersive intervention compared to immersive intervention may emerge. Considering the maximum questionnaire score on each question, the mean obtained in the two interventions indicates that the participant's self-perception of the experience in both sessions was positive (see Supplemental Table 10).

## Discussion

The aim of this study was to analyze the effect of an immersive VR reminiscence session compared to a non-immersive reminiscence session, in terms of engagement in session, BPSD and well-being in people with dementia, using behavioral observation and measurement of physiological metrics during interventions. It was found that the behavioral engagement in session was significantly higher on immersive intervention compared to non-immersive intervention. The results also indicated that apathy on purposeful activity during the session was significantly lower on immersive intervention compared to non-immersive intervention, however apathy on facial expressions during the session was significantly higher on immersive intervention compared to non-immersive intervention. Regarding the physiological analysis, the mean HR in the baseline rest condition was significantly lower than in the exposure condition, in both interventions. As for the other measures evaluated, there were no significant differences between the immersive intervention and non-immersive intervention.

Regarding engagement in the session, the results indicated that behavioral engagement was significantly higher in the immersive intervention than in the non-immersive intervention. This suggests that the immersive intervention would have been more engaging for the participant to watch the video, pointing to locations and attempting to touch or approach objects. This is consistent with scientific research, which indicates that participant engagement during an intervention usually increases with increased immersion in the activity,^37,57^ demonstrating good adherence to these immersive interventions,^37,58^ due to the greater ease in evoking past memories and offering greater opportunity for the participant to explore and interact with the environment through body movements.^33,59^ This result may have been observed due to, combined with the immersiveness of VR, the fact that it was a new and different way of participating in interventions may also have provoked greater interest and engagement.

Interpreting engagement in general and the remaining engagement indicators, it was possible to conclude that there were no significant differences between the immersive and non-immersive intervention. This result is not in line with the scientific literature already cited, which refers that interventions with greater immersiveness tend to be more motivating, thus promoting greater participant engagement.^34,36^ This result can be explained, not only by the small number of participants and number of sessions per interventions, but the fact of carrying out activities using new technologies, such as VR, can be limiting to the participant's participation, due to lack of knowledge and the fact that handling the software can be confusing.^37,60^ Furthermore, greater immersion may also have been a distracting element to the participant's involvement.^57,61^

For BPSD, it was possible to conclude that apathy on purposeful activity was significantly lower in the immersive intervention compared to the non-immersive intervention, indicating greater engagement in the session. This result is in line with scientific evidence that tells us that immersive activities may provide greater interest in the activity, thus also increasing their motivation to engage and intentionally participate in the session.^36,38^ Contrary to this scientific evidence, it was possible to conclude that the apathy demonstrated in facial expressions during the session was significantly higher in the immersive intervention than in the non-immersive intervention. This result can be explained by the fact that the use of HMDs in the facial area may have limited the participant's movements in terms of facial expressions or even limited the researchers’ observation of apathy's facial expressions.

When it comes to the other apathy measures evaluated, through the PEAR, it was possible to conclude that there were no significant differences between the immersive intervention and non-immersive intervention. Handling the non-immersive activity with the computer mouse or the use of VR itself, may have limited the participant's own attention to the activity, which may have influenced the results when it comes to apathy in interventions.^37,58,60^

Regarding pleasure, anger, anxiety, sadness and general alertness, there were no significant differences between immersive and non-immersive intervention. According to scientific evidence, greater immersion could have a greater positive effect on mood, happiness, pleasure, agitation, depressive and anxious symptoms compared to non-immersive activities.^29,36^ This lack of a significative difference can be explained not only by the sample size, but also by the fact that immersive activities can amplify emotions experienced by participants during the intervention, both positive and negative emotions due to the high degree of presence and realism provided by the VR.^57,62^ The fact that there was only one session for each intervention may also have limited the observation of significant differences. Overall, in this study, both interventions showed positive effects on the participants, also making it difficult to conclude significant differences, combined with the possible difficulties of the assessment measures not being sensitive enough to identify differences, especially considering the range of their scores.

Regarding measures of well-being, it was possible to conclude that there were no significant differences between the immersive intervention and non-immersive intervention. Analyzing the results of the OWLS scale, it was possible to verify a tendency for higher levels of happiness and pleasure in the immersive intervention than in the non-immersive intervention, which is in line with studies that report that participants could have higher levels of well-being during activities with immersive interventions, due to the stimulation of memories of autobiographical memories, promoting their sense of identity.^36,63^ The methodology of the instrument used could have been a limitation to observe significant differences, as OWLS is quoted if, in the evaluated period, the first four items are not rated, it is not possible to count the following, even if the person presents these indicators of well-being, which may have influenced these results.^47^ It was also concluded that in both interventions all participants presented themselves attentive, responsive and relaxed during the intervention. This result is in line with scientific evidence that also found that these reminiscence interventions can be an enjoyable and relaxed session, where participants are attentive and focused during the session.^37,58,64^

Regarding physiological measures, it was possible to conclude that mean HR was significantly lower in the baseline rest condition compared to the exposure condition. However, no significant differences were found on the remaining physiological measures. Immersive reminiscence interventions can potentially unbalance the ANS. Due to the fact that it was a reminiscence intervention, capable of promoting the person's interest and causing relaxation and pleasure, there was the possibility of the PNS dominating over the SNS,^65,66^ based on a decrease in the mean HR and increase in SDNN, RMSSD, pNN50, HF and SD1 values.^50,67^ Despite this, reminiscence interventions, being carried out with new technologies, such as VR, can promote interest and engagement in the session, thus increasing the participant's motivation to participate actively,^36,38^ presented by the dominance of the SNS over the PNS, based in the increase in the mean HR, which is in line with the results of this study, and increase in LF, LF/HF ratio values.^65,66^ This dominance of the SNS may also be combined with fear, nervousness or anxiety when carrying out VR activities, due to lack of knowledge about the use and function of these technologies.^37,58^

Regarding SD2 values, although there were no significant differences, there was a tendency for these values ​​to be higher during habituation and exposure conditions compared to resting condition, which is in line with what was previously mentioned, that immersive reminiscence interventions can promote greater motivation to engage in the activity, thus increasing SNS, combined with the increase in mean HR also observed.^31,38^ As this variable is speculative and more reliable over longer-term recordings, it will be necessary to further investigate this relationship with the ANS.

The inability to observe significant differences between the types of intervention may have been due to the positive effects caused by both interventions, evidenced by the participant's own self-perception of the experience.

In terms of the participant's self-perception of the experience, although the results indicated that there were no significant differences, it was possible to observe a tendency to enjoy the immersive intervention more than the non-immersive intervention. This result is in line with scientific evidence that says that immersive intervention is usually more appreciated, more satisfying and interesting for participants than non-immersive intervention, with good adherence and participation.^34,36^ Studies also support the safety and feasibility of using VR to promote autobiographical memory activities.^29,31^ It was also possible to observe the tendency to see the video presented in the non-immersive intervention better than in the immersive one. The comfort factor of the HMD in conjunction with the participants’ prescription glasses, may account for this outcome.

This study has several strengths, notably its exploration of the under-researched area of VR reminiscence for individuals with dementia, focusing on engagement, BPSD, and well-being. The inclusion of physiological data during sessions is a significant contribution, as it is often overlooked yet essential for evaluation. The study utilized diverse instruments targeting various aspects of each variable, allowing for comprehensive assessment of the interventions and their effects on participants. Observation-based measures were particularly relevant given the communication challenges associated with dementia. Additionally, confirming participants’ life stories with caregivers to select appropriate videos enhanced the intervention's relevance, acknowledging the memory deficits present in some participants. The personalized nature of the interventions likely fostered greater engagement and impact.^26,68^

However, the study also faced limitations. The small sample size may have affected the power and significance of statistical analyses, compounded by the limited representation of only two male participants, which restricts the generalizability of the findings. Unconscious bias was another concern, as researchers conducting the interventions were aware of the study's hypotheses. The static seating arrangement during the immersive intervention aimed to mitigate adverse effects, such as nausea or fall risk, but may have restricted participants’ ability to engage fully with the 360° video, potentially diminishing the immersive experience. Conducting only one session of each intervention may have influenced outcomes, as participant condition on the intervention day could vary. Additionally, the use of a computer mouse in the non-immersive session posed challenges for some participants, affecting their engagement. Variability in recognition of the habituation video may have led to differences in participation levels, impacting results. The need to remove the HMD between habituation and exposure periods may have affected physiological analyses, while the different progression of dementia stages throughout the participants could also have influenced outcomes across the two interventions, as there may have been a transition effect from the first intervention to the second intervention. Finally, the absence of a passive control group or physical reminiscence activities may also represent a limitation, as it prevents definitive attribution of the observed changes to the intervention.

## Conclusion

In this study it was concluded that immersive intervention significantly enhanced engagement and reduced activity-related apathy compared to non-immersive intervention. However, no significant differences were found in well-being and the remaining BSPD, despite the results suggesting a tendency for a greater positive effect in the immersive intervention, such as happiness, pleasure and enjoyment. These findings highlight the potential of using technologies like VR and computers to improve adherence and engagement in dementia intervention programs, given their increasing accessibility and safety.

Future research should involve larger, more balanced samples to allow for robust statistical analyses and generalization of results. The possibility of introducing external evaluators or blinding procedures, having an impact on blinding bias, should be explored in future studies. Exploring the use of swivel chairs for safe viewing of 360° videos may enhance participant engagement with significant locations. The effectiveness of presenting the habituation and exposure video in the same video without interruptions and without having to remove the HMD, enhancing the effectiveness of the habituation period and transition to the exposure period, should also be studied. In non-immersive sessions, simplifying video interactions—such as showing the 360° video on the computer automatically or on a touch screen—should be considered. Furthermore, it would be important to carry out studies with a greater number of sessions for each of the interventions, reducing the external influences that may occur on each participant and could provide better insights into engagement, BPSD, and well-being. If possible, always hold the sessions at the same time and place, without disrupting the participant's daily routine. The best conditions for immersive interventions, including session length, frequency, and the utilization of various VR hardware should also be explored. Future studies should also ensure that habituation videos are universally recognized by participants, potentially by using familiar settings. Evaluating the effectiveness of various washout periods will be crucial for this population. Future studies should also include long-term follow-up assessments to evaluate the sustained effects of immersive VR. Moreover, assessing the impact of family involvement in interventions could deepen engagement with the memories associated with the presented locations. Investigating these interventions in more advanced stages of dementia or evaluating these outcomes and exploring intervention strategies in home or hospital settings, warrants consideration. The portability and flexibility of VR-based interventions provide an opportunity to provide adequate reminiscence sessions for people with dementia across several settings and disease stages.

Furthermore, continued use of observational tools and physiological measures will enrich data collection, particularly for participants with difficulties in emotional expression. Future studies should consider using assessment tools compatible with immersive devices, such as facial recognition technology or equipment that allows greater freedom of facial movement. Future studies might also explore additional physiological metrics, such as galvanic skin response pupillometry, and neural markers (e.g., functional near-infrared spectroscope or electroencephalography) to better understand the biological underpinnings of VR-related emotional and arousal responses.

In summary, incorporating immersive virtual reality as a non-pharmacological therapy tool presents significant clinical potential, and its feasibility in real-world clinical settings warrants careful consideration, particularly in terms of accessibility, cost-effectiveness, and integration into existing treatment protocols. Such research aims to provide safe, beneficial activities that improve the quality of life for individuals with dementia and their caregivers.

## Supplemental Material

sj-docx-1-alz-10.1177_13872877251371236 - Supplemental material for Effects of an immersive virtual reality reminiscence intervention on engagement, behavioral and psychological symptoms, and well-being of people with dementia: A randomized crossover trial Supplemental material, sj-docx-1-alz-10.1177_13872877251371236 for Effects of an immersive virtual reality reminiscence intervention on engagement, behavioral and psychological symptoms, and well-being of people with dementia: A randomized crossover trial by Miguel Pereira, Cláudia Leite, Carlos Campos and Tiago Coelho in Journal of Alzheimer's Disease

## References

[1] Desai AK Schwartz L Grossberg GT . Behavioral disturbance in dementia. Curr Psychiatry Rep 2012; 14: 298–309. 22644311 10.1007/s11920-012-0288-5

[2] Leng M Han S Sun Y , et al. Identifying care problem clusters and core care problems of older adults with dementia for caregivers: a network analysis. Front Public Health 2023; 11: 1195637. 37637827 10.3389/fpubh.2023.1195637 PMC10449331

[3] Bessey LJ Walaszek A . Management of behavioral and psychological symptoms of dementia. Curr Psychiatry Rep 2019; 21: 66. 31264056 10.1007/s11920-019-1049-5

[4] Savva GM Zaccai J Matthews FE , et al. Prevalence, correlates and course of behavioural and psychological symptoms of dementia in the population. Br J Psychiatry 2009; 194: 212–219. 19252147 10.1192/bjp.bp.108.049619

[5] Wetzels RB Zuidema SU de Jonghe JFM , et al. Determinants of quality of life in nursing home residents with dementia. Dement Geriatr Cogn Disord 2010; 29: 189–197. 20215750 10.1159/000280437

[6] Cooper C Mukadam N Katona C , et al. Systematic review of the effectiveness of non-pharmacological interventions to improve quality of life of people with dementia. Int Psychogeriatrics 2012; 24: 856–870. 10.1017/S1041610211002614 22244371

[7] World Health Organization . Dementia, https://www.who.int/news-room/fact-sheets/detail/dementia (2003).

[8] Li W Xu X Wu F , et al. Comparative efficacy of non-pharmacological interventions on behavioural and psychological symptoms in elders with dementia: a network meta-analysis. Nurs Open 2021; 8: 2922–2931. 34472717 10.1002/nop2.1049 PMC8510770

[9] National Institute for Health and Care Excellence . Dementia: assessment, management and support for people living with dementia and their carers. *NICE Guidelines*, www.nice.org.uk/guidance/ng97 (2018).

[10] Seppala LJ Wermelink AMAT de Vries M , et al. Fall-risk-increasing drugs: a systematic review and meta-analysis: II. Psychotropics. J Am Med Dir Assoc 2018; 19: 371.e11–371.e17. 10.1016/j.jamda.2017.12.098 29402652

[11] Gill SS Bronskill SE Normand SL , et al. Antipsychotic drug use and mortality in older adults with dementia. Ann Intern Med 2007; 146: 775–786. 17548409 10.7326/0003-4819-146-11-200706050-00006

[12] Abraha I Rimland JM Trotta FM , et al. Systematic review of systematic reviews of non-pharmacological interventions to treat behavioural disturbances in older patients with dementia. The SENATOR-OnTop series. BMJ Open 2017; 7: e012759. 10.1136/bmjopen-2016-012759 PMC5372076 28302633

[13] Dyer SM Harrison SL Laver K , et al. An overview of systematic reviews of pharmacological and non-pharmacological interventions for the treatment of behavioral and psychological symptoms of dementia. Int Psychogeriatrics 2018; 30: 295–309. 10.1017/S1041610217002344 29143695

[14] Kuiper JS Zuidersma M Oude Voshaar RC , et al. Social relationships and risk of dementia: a systematic review and meta-analysis of longitudinal cohort studies. Ageing Res Rev 2015; 22: 39–57. 25956016 10.1016/j.arr.2015.04.006

[15] Kishita N Backhouse T Mioshi E . Nonpharmacological interventions to improve depression, anxiety, and quality of life (QoL) in people with dementia: an overview of systematic reviews. J Geriatr Psychiatry Neurol 2020; 33: 28–41. 31203712 10.1177/0891988719856690

[16] Brosch T Scherer K Grandjean D , et al. The impact of emotion on perception, attention, memory, and decision-making. Swiss Med Wkly 2013; 143: w13786. 10.4414/smw.2013.13786 23740562

[17] Vuijk JGJ Klein Brinke J Sharma N . Utilising emotion monitoring for developing music interventions for people with dementia: a state-of-the-art review. Sensors (Basel) 2023; 23: 5834. 37447684 10.3390/s23135834 PMC10347021

[18] Yanagida N Yamaguchi T Matsunari Y . Evaluating the impact of reminiscence therapy on cognitive and emotional outcomes in dementia patients. J Pers Med 2024; 14: 629. 38929850 10.3390/jpm14060629 PMC11204563

[19] Norris A . Reminiscence with elderly people. London: Winslow Press, 1986.

[20] Kiernat J . The use of life review activity with confused nursing home residents. Am J Occup Ther 1979; 33: 306–310. 474339

[21] Woods B O’Philbin L Farrell EM , et al. Reminiscence therapy for dementia. Cochrane Database Syst Rev 2018; 2018: CD001120. 10.1002/14651858.CD001120.pub3 PMC6494367 29493789

[22] Wu Y Xu H Sui X , et al. Effects of group reminiscence interventions on depressive symptoms and life satisfaction in older adults with intact cognition and mild cognitive impairment: a systematic review. Arch Gerontol Geriatr 2023; 114: 105103. 37354738 10.1016/j.archger.2023.105103

[23] Lee KH Lee JY Kim B . Person-centered care in persons living with dementia: a systematic review and meta-analysis. Gerontologist 2022; 62: e253–e264. 10.1093/geront/gnaa207 PMC9019632 33326573

[24] Jin B Xv Y Zhang B , et al. Comparative efficacy and acceptability of treatments for depressive symptoms in cognitive impairment: a systematic review and Bayesian network meta-analysis. Front Aging Neurosci 2022; 14: 1037414. 36578447 10.3389/fnagi.2022.1037414 PMC9790988

[25] Lök N Bademli K Selçuk-Tosun A . The effect of reminiscence therapy on cognitive functions, depression, and quality of life in Alzheimer patients: randomized controlled trial. Int J Geriatr Psychiatry 2019; 34: 47–53. 30246408 10.1002/gps.4980

[26] Huang HC Chen YT Chen PY , et al. Reminiscence therapy improves cognitive functions and reduces depressive symptoms in elderly people with dementia: a meta-analysis of randomized controlled trials. J Am Med Dir Assoc 2015; 16: 1087–1094. 26341034 10.1016/j.jamda.2015.07.010

[27] Chapoulie E Guerchouche R Petit PD , et al. Reminiscence therapy using image-based rendering in VR. In: 2014 IEEE virtual reality (VR). New York: IEEE, 2014, pp.45–50.

[28] Rose V Stewart I Jenkins KG , et al. A scoping review exploring the feasibility of virtual reality technology use with individuals living with dementia. In: 28th international conference on artificial reality and telexistence, 23rd eurographics symposium on virtual environments, 2018, pp.131–139. 10.2312/egve.20181325

[29] Rose V Stewart I Jenkins KG , et al. Bringing the outside in: the feasibility of virtual reality with people with dementia in an inpatient psychiatric care setting. Dementia 2021; 20: 106–129. 31510801 10.1177/1471301219868036

[30] Vergara D Rubio M Lorenzo M . On the design of virtual reality learning environments in engineering. Multimodal Technol Interact 2017; 1: 11.

[31] Kim O Pang Y Kim JH . The effectiveness of virtual reality for people with mild cognitive impairment or dementia: a meta-analysis. BMC Psychiatry 2019; 19: 219. 31299921 10.1186/s12888-019-2180-x PMC6626425

[32] Appel L Kisonas E Appel E , et al. Administering virtual reality therapy to manage behavioral and psychological symptoms in patients with dementia admitted to an acute care hospital: results of a pilot study. JMIR Form Res 2021; 5: e22406. 10.2196/22406 PMC7889418 33533720

[33] Huang LC Yang YH . The long-term effects of immersive virtual reality reminiscence in people with dementia: longitudinal observational study. JMIR Serious Games 2022; 10: e36720. 10.2196/36720 PMC9361147 35877169

[34] Saredakis D Keage HA Corlis M , et al. Using virtual reality to improve apathy in residential aged care: mixed methods study. J Med Internet Res 2020; 22: e17632. 10.2196/17632 PMC7380990 32469314

[35] Coelho T Marques C Moreira D , et al. Promoting reminiscences with virtual reality headsets: a pilot study with people with dementia. Int J Environ Res Public Health 2020; 17: 9301. 33322679 10.3390/ijerph17249301 PMC7763810

[36] D’Cunha NM Nguyen D Naumovski N , et al. A mini-review of virtual reality-based interventions to promote well-being for people living with dementia and mild cognitive impairment. Gerontology 2019; 65: 430–440. 31108489 10.1159/000500040

[37] White PJ Moussavi Z . Neurocognitive treatment for a patient with Alzheimer’s disease using a virtual reality navigational environment. J Exp Neurosci 2016; 10: 129–135. 27840579 10.4137/JEN.S40827 PMC5102253

[38] Saredakis D Keage HA Corlis M , et al. The effect of reminiscence therapy using virtual reality on apathy in residential aged care: multisite nonrandomized controlled trial. J Med Internet Res 2021; 23: e29210. 10.2196/29210 PMC8491119 34542418

[39] Khirallah Abd El Fatah N Abdelwahab Khedr M Alshammari M , et al. Effect of immersive virtual reality reminiscence versus traditional reminiscence therapy on cognitive function and psychological well-being among older adults in assisted living facilities: a randomized controlled trial. Geriatr Nurs (Minneap) 2024; 55: 191–203. 10.1016/j.gerinurse.2023.11.010 38007908

[40] Dwan K Li T Altman DG , et al. CONSORT 2010 Statement: extension to randomised crossover trials. Br Med J 2019; 366: l4378. 10.1136/bmj.l4378 PMC6667942 31366597

[41] Holstila E Vallittu A Ranto S , et al. World medical association declaration of Helsinki. JAMA 2013; 310: 2191. 24141714 10.1001/jama.2013.281053

[42] Reisberg B Ferris S De Leon M , et al. The global deterioration scale for assessment of primary degenerative dementia. Am J Psychiatry 1982; 139: 1136–1139. 7114305 10.1176/ajp.139.9.1136

[43] Barreto J Leuschner A Santos F , et al. Escalas e Testes Na Demência [Scales and Tests in Dementia]. 2nd ed. Condeixa, Coimbra, Portugal: Grupo de Estudos de Envelhecimento Cerebral e Demências [Study Group on Brain Aging and Dementia], 2008.

[44] Jones C Sung B Moyle W . Engagement of a person with dementia scale: establishing content validity and psychometric properties. J Adv Nurs 2018; 74: 2227–2240. 10.1111/jan.13717 29772602

[45] Jao YL Algase DL Specht JK , et al. Developing the person–environment apathy rating for persons with dementia. Aging Ment Health 2016; 20: 861–870. 25984756 10.1080/13607863.2015.1043618

[46] Lawton M Van Haitsma K Klapper J . Observed Emotion Rating Scale, https://abramsonseniorcare.org/media/1199/observed-emotion-rating-scale.pdf (1999).

[47] Madsø KG Pachana NA Nordhus IH . Development of the observable well-being in living with dementia-scale. Am J Alzheimers Dis Other Demen 2023; 38: 15333175231171990. 37269060 10.1177/15333175231171990 PMC10624086

[48] Kourtis LC Regele OB Wright JM , et al. Digital biomarkers for Alzheimer’s disease: the mobile/wearable devices opportunity. NPJ Digit Med 2019; 2: 9. 31119198 10.1038/s41746-019-0084-2 PMC6526279

[49] Perna G Riva A Defillo A , et al. Heart rate variability: can it serve as a marker of mental health resilience? J Affect Disord 2020; 263: 754–761. 31630828 10.1016/j.jad.2019.10.017

[50] Shaffer F Ginsberg JP . An overview of heart rate variability metrics and norms. Front Public Health 2017; 5: 258. 29034226 10.3389/fpubh.2017.00258 PMC5624990

[51] Laborde S Mosley E Thayer JF . Heart rate variability and cardiac vagal tone in psychophysiological research – recommendations for experiment planning, data analysis, and data reporting. Front Psychol 2017; 8: 213. 28265249 10.3389/fpsyg.2017.00213 PMC5316555

[52] Nayak SK Pradhan B Mohanty B , et al. A review of methods and applications for a heart rate variability analysis. Algorithms 2023; 16: 433.

[53] Shaffer F McCraty R Zerr CL . A healthy heart is not a metronome: an integrative review of the heart’s anatomy and heart rate variability. Front Psychol 2014; 5: 1040. 25324790 10.3389/fpsyg.2014.01040 PMC4179748

[54] Gomes P Margaritoff P Plácido da Silva H . pyHRV: development and evaluation of an open-source python toolbox for heart rate variability (HRV). In: Int conf electr electron comput eng, 2019, pp.822–828.

[55] Gomes P . pyHRV – OpenSource Python Toolbox for Heart Rate Variability Documentation Release 0.4 (2022).

[56] Gignac G. *How2statsbook (Online Edition 2)* (2023).

[57] Lu Z Wang W Yan W , et al. The application of fully immersive virtual reality on reminiscence interventions for older adults: scoping review. JMIR Serious Games 2023; 11: e45539. 10.2196/45539 PMC10589832 37801360

[58] Mendez MF Karve S Jimenez E . Virtual reality for the assessment of frontotemporal dementia, a feasibility study. Disabil Rehabil Assist Technol 2015; 10: 160–164. 24524440 10.3109/17483107.2014.889230

[59] Kirk M Rasmussen KW Overgaard SB , et al. Five weeks of immersive reminiscence therapy improves autobiographical memory in Alzheimer’s disease. Memory 2019; 27: 441–454. 30198380 10.1080/09658211.2018.1515960

[60] Akhter R Sun W Quevedo AJU , et al. Perceived barriers and solutions identified by healthcare professionals in utilizing web-based reminiscence therapy to support dementia care during the pandemic. Aging Clin Exp Res 2023; 35: 2843–2846. 37581860 10.1007/s40520-023-02520-w

[61] Richardson A Seals CD Garza KB , et al. Non-immersive vs. immersive: the difference in empathy, user engagement, and user experience when simulating the daily life of rheumatoid arthritis patients. In: Duffy VG (ed) Digital human modeling and applications in health, safety, ergonomics and risk management. HCII 2023. Lecture Notes in Computer Science, vol. 14029. Cham: Springer, 2023, pp.562–575.

[62] Somarathna R Bednarz T Mohammadi G . Virtual reality for emotion elicitation – a review. IEEE Trans Affect Comput 2022; 14: 2626–2645.

[63] Park K Lee S Yang J , et al. A systematic review and meta-analysis on the effect of reminiscence therapy for people with dementia. Int Psychogeriatrics 2019; 31: 1581–1597. 10.1017/S1041610218002168 30712519

[64] Chaze F Hayden L Azevedo A , et al. Virtual reality and well-being in older adults: results from a pilot implementation of virtual reality in long-term care. J Rehabil Assist Technol Eng 2022; 9: 205566832110723. 10.1177/20556683211072384 PMC8832624 35154808

[65] Aganov S Nayshtetik E Nagibin V , et al. Pure purr virtual reality technology: measuring heart rate variability and anxiety levels in healthy volunteers affected by moderate stress. Arch Med Sci 2020; 18: 336–343. 35316901 10.5114/aoms.2020.93239 PMC8924843

[66] Hsieh CH Li D . Understanding how virtual reality forest experience promote physiological and psychological health for patients undergoing hemodialysis. Front Psychiatry 2022; 13: 1007396. 36590601 10.3389/fpsyt.2022.1007396 PMC9794622

[67] Appel L Appel E Bogler O , et al. Older adults with cognitive and/or physical impairments can benefit from immersive virtual reality experiences: a feasibility study. Front Med (Lausanne) 2020; 6: 329. 32010701 10.3389/fmed.2019.00329 PMC6974513

[68] Saragih ID Tonapa SI Yao C , et al. Effects of reminiscence therapy in people with dementia: a systematic review and meta-analysis. J Psychiatr Ment Health Nurs 2022; 29: 883–903. 35348260 10.1111/jpm.12830

