# Supplementary material for: Effects of an immersive virtual reality reminiscence intervention on engagement, behavioral and psychological symptoms, and well-being of people with dementia: A randomized crossover trial
Source: J Alzheimers Dis. 2025 Aug 29;107(4):1517–29. doi: 10.1177/13872877251371236 (PMC12495103; doi:10.1177/13872877251371236)
Supplement: sj-docx-1-alz-10.1177_13872877251371236 - Supplemental material for Effects of an immersive virtual reality reminiscence intervention on engagement, behavioral and psychological symptoms, and well-being of people with dementia: A randomized crossover trial [file sj-docx-1-alz-10.1177_13872877251371236.docx]

**Supplemental Material**

**Effects of an immersive virtual reality reminiscence intervention on engagement, behavioral and psychological symptoms, and well-being of people with dementia: a randomized crossover trial**

**Supplemental Table 1.** Comparison of the immersive and non-immersive intervention according to the EPWDS scores.

| Mean ± Standard deviation | | | |
| --- | --- | --- | --- |
|  | **Immersive intervention** | **Non-immersive intervention** | ***p*** |
| EPWDS | 43.75 ± 4.36 | 43.05 ± 4.21 | 0.303^a^ |

^a^values ​​obtained through the T test for paired samples.

**Supplemental Table 2.** Comparison of the immersive and non-immersive intervention according to the OERS scores.

|  | Mean ± Standard deviation | | | *p* |
| --- | --- | --- | --- | --- |
|  | **Immersive intervention** | **Non-immersive intervention** |  | |
| Pleasure | 3.80±1.06 | 3.75±1.02 | 0.803^b^ | |
| Anger | 1.00±0.00 | 1.00±0.00 | 1.000^b^ | |
| Anxiety/fear | 1.05±0.22 | 1.10±0.31 | 0.317^b^ | |
| Sadness | 1.50±0.76 | 1.55±0.89 | 0.705^b^ | |
| General alertness | 4.60±0.50 | 4.60±0.50 | 1.000^b^ | |

^b^value obtained using the Wilcoxon signed-rank test.

**Supplemental Table 3.** Comparison of the immersive and non-immersive intervention according to the OWLS scores.

|  | Mean ± Standard deviation | | | *p* | |
| --- | --- | --- | --- | --- | --- |
|  | **Immersive intervention** | **Non-immersive intervention** |  | |  |
| Attention | 10.00±0.00 | 10.00±0.00 | 1.000^b^ | |  |
| Initiative/responsiveness | 10.00±0.00 | 10.00±0.00 | 1.000^b^ | |  |
| Relaxation | 10.00±0.00 | 10.00±0.00 | 1.000^b^ | |  |
| Happiness | 9.10±1.65 | 8.70±2.13 | 0.382^b^ | |  |
| Pleasure | 5.90±2.69 | 5.05±2.89 | 0.245^b^ | |  |
| Identity expressions | 6.50±2.40 | 6.30±2.49 | 0.795^b^ | |  |
| Mastery | 3.70±2.43 | 3.75±2.47 | 0.745^b^ | |  |
| Relationships | 3.05±2.37 | 3.30±2.39 | 0.293^b^ | |  |

^b^value obtained using the Wilcoxon signed-rank test.

**Supplemental Table 4.** Descriptive statistics for physiological data results.

|  |  | Immersive intervention | | | | | | Non-immersive intervention | | | | | |
| --- | --- | --- | --- | --- | --- | --- | --- | --- | --- | --- | --- | --- | --- |
|  |  | Resting | | Habituation | | Exposure | | Resting | | Habituation | | Exposure | |
|  |  | M | SD | M | SD | M | SD | M | SD | M | SD | M | SD |
| Time Domain | Mean HR* (bpm)  SDNN* (ms)  RMSSD* (ms)  pNN50 (%) | 67.75  54.91  68.15  21.95 | 2.71  11.07  14.74  5.79 | 70.29  64.75  74.56  20.34 | 2.43  12.50  16.70  5.78 | 70.16  56.45  68.14  22.56 | 2.61  8.74  14.65  6.73 | 67.35  47.02  57.42  28.12 | 2.55  8.83  11.97  7.78 | 70.80  62.09  77.79  29.47 | 2.85  9.25  13.11  7.64 | 69.41  58.61  72.07  27.60 | 2.16  12.36  16.16  7.12 |
| Frequency Domain | Peak Frequencies on LF (Hz)  Peak Frequencies on HF (Hz)  Absolute Powers on LF** (ms^2)  Absolute Powers on HF** (ms^2)  Total Power*** (ms^2)  LF/HF Ratio**** (-) | 0.08  0.28  439.79  1312.25  2882.99  0.81 | 0.01  0.2  191.58  709.72  1198.18  0.37 | 0.08  0.26  936.82  2616.25  3073.20  0.64 | 0.01  0.2  440.69  1300.45  1027.44  0.16 | 0.07  0.28  643.60  884.48  2436.12  0.73 | 0.01  0.2  239.73  388.28  720.09  0.21 | 0.07  0.29  686.25  629.62  2744.16  0.54 | 0.01  0.2  562.64  194.71  1017.57  0.14 | 0.08  0.28  709.36  1231.55  4065.57  0.68 | 0.01  0.2  337.29  398.55  1314.27  0.22 | 0.07  0.28  766.43  599.29  4100.21  0.65 | 0.01  0.2  458.94  219.08  2076.64  0.16 |
| Nonlinear methods | SD1* (ms)  SD2* (ms)  SD2/SD1***** (-) | 48.18  57.29  1.42 | 10.43  12.67  0.21 | 52.72  72.43  1.67 | 11.81  13.82  0.22 | 48.18  61.61  1.53 | 10.36  7.79  0.18 | 40.60  49.88  1.41 | 8.46  9.87  0.19 | 55.01  66.14  1.55 | 9.27  10.16  0.21 | 50.96  63.29  1.46 | 11.43  13.79  0.19 |

*n = 17 (3 outliers); **n = 15 (5 outliers); ***n = 16 (4 outliers); ****n = 14 (6 outliers); *****n = 19 (1 outlier).

**Supplemental Table 5.** Inferential statistics from the repeated-measures ANOVA on Time Domain results.

|  |  | F | *p* | partial η2 |
| --- | --- | --- | --- | --- |
| Mean HR* (bpm) | Intervention  Condition  Condition*Intervention interaction | 0.026  4.333  0.279 | 0.875^c^  0.043^d^  0.641^d^ | 0.002  0.213  0.017 |
| SDNN* (ms) | Intervention  Condition  Condition*Intervention interaction | 0.069  2.501  0.256 | 0.796^c^  0.098^c^  0.776^c^ | 0.004  0.135  0.016 |
| RMSSD* (ms) | Intervention  Condition  Condition*Intervention interaction | 0.007  1.729  0.458 | 0.935^c^  0.194^c^  0.637^c^ | 0.000  0.098  0.028 |
| pNN50 (%) | Intervention  Condition  Condition*Intervention interaction | 1.466  0.006  0.611 | 0.241^c^  0.994^c^  0.548^c^ | 0.072  0.000  0.031 |

*n = 17 (3 outliers)

^c^value obtained through the two-way ANOVA test

^d^value obtained through the two-way ANOVA test (Huynh-Feldt correction)

**Supplemental Table 6.** Post-hoc pairwise comparisons between the conditions effect for Mean HR.

|  | Mean HR* | *p* |
| --- | --- | --- |
|  | Mean ± Standard deviation |  |
| Habituation - Resting | 3.00±1.42 | 0.154^e^ |
| Exposure - Habituation | -0.76±0.91 | 1.000^e^ |
| Exposure - Resting | 2.24±0.72 | 0.020^e^ |

*n = 17 (3 outliers)

^e^value obtained through the post-hoc pairwise Bonferroni correction

**Supplemental Table 7.** Inferential statistics from the repeated-measures ANOVA on Frequency Domain results.

|  |  | F | *p* | partial η2 |
| --- | --- | --- | --- | --- |
| Peak Frequencies on LF (Hz) | Intervention  Condition  Condition*Intervention interaction | 0.112  0.857  0.076 | 0. 741^c^  0.433^d^  0.927^d^ | 0.006  0.043  0.004 |
| Peak Frequencies on HF (Hz) | Intervention  Condition  Condition*Intervention interaction | 0.297  0.480  0.182 | 0.592^c^  0.623^c^  0.834^c^ | 0.015  0.025  0.010 |
| Absolute Powers on LF** (ms^2) | Intervention  Condition  Condition*Intervention interaction | 0.029  0.465  0.231 | 0.866^c^  0.633^c^  0.672^d^ | 0.002  0.032  0.016 |
| Absolute Powers on HF** (ms^2) | Intervention  Condition  Condition*Intervention interaction | 1.386  2.493  0.449 | 0.259^c^  0.130^d^  0.530^f^ | 0.090  0.151  0.031 |
| Total Power*** (ms^2) | Intervention  Condition  Condition*Intervention interaction | 0.460  0.295  0.471 | 0.508^c^  0.679^d^  0.629^c^ | 0.030  0.019  0.030 |
| LF/HF Ratio**** (-) | Intervention  Condition  Condition*Intervention interaction | 0.272  0.015  0.739 | 0.611^c^  0.985^c^  0.488^c^ | 0.020  0.001  0.054 |

**n = 15 (5 outliers); ***n = 16 (4 outliers); ****n = 14 (6 outliers).

^c^value obtained through the two-way ANOVA test

^d^value obtained through the two-way ANOVA test (Huynh-Feldt correction)

^f^value obtained through the two-way ANOVA test (Greenhouse-Geisser correction)

**Supplemental Table 8.** Inferential statistics from the repeated-measures ANOVA on Non-linear methods results.

|  |  | F | *p* | partial η2 |
| --- | --- | --- | --- | --- |
| SD1* (ms) | Intervention  Condition  Condition*Intervention interaction | 0.007  1.729  0.458 | 0.935^c^  0.194^d^  0.637^d^ | 0.000  0.098  0.028 |
| SD2* (ms) | Intervention  Condition  Condition*Intervention interaction | 0.124  2.858  0.171 | 0.729^c^  0.072^c^  0.843^c^ | 0.008  0.152  0.011 |
| SD2/SD1***** (-) | Intervention  Condition  Condition*Intervention interaction | 0.177  0.970  0.086 | 0.679^c^  0.389^c^  0.918^c^ | 0.010  0.051  0.005 |

*n = 17 (3 outliers); *****n = 19 (1 outlier).

^c^value obtained through the two-way ANOVA test

**Supplemental Table 9.** Descriptive statistics from the participant's self-perception of the experience questionnaire.

|  |  | n (%) | | | | |
| --- | --- | --- | --- | --- | --- | --- |
|  |  | 1 | 2 | 3 | 4 | 5 |
|  |  | “I didn't like it very much” | “I didn't like it.” | “I didn't like it, nor did I like it.” | “I liked it” | “I liked it a lot.” |
| "Did you like the experience?" | Immersive | 0 (0) | 0 (0) | 0 (0) | 4 (20) | 16 (80) |
|  | Non-immersive | 0 (0) | 0 (0) | 1 (5) | 6 (30) | 13 (65) |
|  |  | “Very bad.” | “Bad.” | “Neither bad nor good.” | “Good.” | “Very Good.” |
| "How was the experience?" | Immersive | 0 (0) | 0 (0) | 2 (10) | 7 (35) | 11 (55) |
|  | Non-immersive | 0 (0) | 0 (0) | 1 (5) | 11 (55) | 8 (40) |
|  |  | “Very Unmotivated.” | “Unmotivated.” | “Neither unmotivated nor motivated.” | “Motivated.” | “Very motivated.” |
| "How motivated are you to do this activity again?" | Immersive | 0 (0) | 0 (0) | 6 (30) | 7 (35) | 7 (35) |
|  | Non-immersive | 0 (0) | 2 (10) | 2 (10) | 12 (60) | 4 (20) |
|  |  | “Very disinterested.” | “Disinterested.” | “Neither disinterested nor interested.” | “Interested.” | “Very interested.” |
| "Interested in seeing other places?" | Immersive | 0 (0) | 0 (0) | 5 (25) | 11 (55) | 4 (20) |
|  | Non-immersive | 0 (0) | 1 (5) | 4 (20) | 8 (40) | 7 (35) |
|  |  | “Very bad.” | “Bad.” | “Neither bad nor good.” | “Good.” | “Very Good.” |
| "How well did you manage to watch the video?" | Immersive | 0 (0) | 0 (0) | 4 (20) | 9 (45) | 7 (35) |
|  | Non-immersive | 0 (0) | 0 (0) | 0 (0) | 10 (50) | 10 (50) |
|  |  | “Very uncomfortable.” | “Uncomfortable.” | “Neither uncomfortable nor comfortable.” | “Comfortable.” | “Very comfortable.” |
| "How comfortable was the activity?" | Immersive | 0 (0) | 0 (0) | 1 (5) | 9 (45) | 10 (50) |
|  | Non-immersive | 0 (0) | 0 (0) | 0 (0) | 9 (45) | 11 (55) |

**Supplemental Table 10.** Comparison of the immersive and non-immersive intervention according to the participant's self-perception of the experience questionnaire.

|  | Mean ± Standard deviation | | *p* | |
| --- | --- | --- | --- | --- |
|  | **Immersive intervention** | **Non-immersive intervention** |  |  |
| "Did you like the experience?" | 4.80±0.41 | 4.60±0.60 | 0.102^b^ |  |
| "How was the experience?" | 4.45±0.69 | 4.35±0.59 | 0.414^b^ |  |
| "How motivated are you to do this activity again?" | 4.05±0.83 | 3.90±0.85 | 0.439^b^ |  |
| "Interested in seeing other places?" | 3.95±0.69 | 4.05±0.89 | 0.627^b^ |  |
| "How well did you manage to watch the video?" | 4.15±0.75 | 4.50±0.51 | 0.070^b^ |  |
| "How comfortable was the activity?" | 4.45±0.61 | 4.55±0.51 | 0.480^b^ |  |

^b^value obtained using the Wilcoxon signed-rank test.
